# Supplementary material for: Measuring relative opinion from location-based social media: A case study of the 2016 U.S. presidential election
Source: PLoS One. 2020 May 22;15(5):e0233660. doi: 10.1371/journal.pone.0233660 (PMC7244148; doi:10.1371/journal.pone.0233660)
Supplement: S1 Appendix — (DOCX) [file pone.0233660.s001.docx]

**S1 Appendix**

**A. The procedure of creating training data**

The procedure used in the section ‘Data collection and training data for opinion identification’ is described as follows. It includes five steps:

1) Identifying seed hashtags. Among the most frequent hashtags, we identify four hashtags, each one representing a different opinion category: #maga for pro-Trump (the abbreviation of the official Trump campaign slogan: Make America Great Again), #imwithher for pro-Hillary (the official Clinton campaign slogan), #nevertrump for anti-Trump, and #neverhillary for anti-Clinton.

2) Building co-occurrence networks of hashtags. The hashtag co-occurrence network $H(V,E)$ is then constructed, where the set of vertices $v_{i}\in V$ represents hashtags, and an edge $e_{ij}$ is drawn between $v_{i}$ and $v_{j}$ if they appear together in a tweet. The resulting graph has 108,600 vertices and 881,344 edges.

3) Computing similarity networks of hashtags. With the constructed $H(V,E)$, we test the significance of each edge $e_{ij}$ [1]. Specifically, the probability $p_{ij}$ (p-value of the null hypothesis) to observe the corresponding number of co-occurrences $k$ by chance is computed given the number of occurrences $n_{i}$ and $n_{j}$ of the vertices $v_{i}$ and $v_{j}$, and the total number of tweets $N$ (Equation 1). Only significant edges satisfying $p_{ij}< p_{o}$, where $p_{o}={10}^{-6}$, are kept, effectively filtering out spurious relations between hashtags. Then, a weight $s_{ij}$ is assigned to the edge $e_{ij}$ (based on Equation 2) representing the significance of the similarity between two hashtags. Retaining only significant similarity linkages reduces the graph to 108,600 vertices and 51,633 edges, which comprises a similarity network of hashtags.

$p_{ij}\left( k \right)=\prod_{m=0}^{n_{j}-k-1} \left( 1-\frac{n_{i}}{N-m} \right)\prod_{m=0}^{k-1} \frac{(n_{i}-m)(n_{j}-m)}{(N-n_{j}+k-m)(k-m)}$ (1)

$s_{ij}=log(\frac{p_{o}}{p_{ij}})$ (2)

4) Classifying hashtags in the similarity network. The similarity network is then used to discover and classify hashtags that are significantly similar to the seed hashtags as in [2,3]. The main idea behind the classification of hashtags is that a vertex $v_{i}$ determines its label, denoting the class to which it belongs, based on the labels of its neighbors, defined as vertices that have edges with $v_{i}$. It is assumed that each $v_{i}$ in the network chooses to obtain the label carried by the largest number of its neighbors, with ties broken uniformly randomly. Formally, if $C_{1}, \ldots, C_{j}$ are the labels that are currently active in the network and $d_{i}^{C_{j}}$ is the number of neighbors $v_{i}$ has with label $C_{j}$, then every $v_{i}$ keeps acquiring its label until for each $v_{i}$,

$If v_{i} has label C_{m}, then d_{i}^{C_{m}}\geq d_{i}^{C_{j}}$ (3)

5) Network pruning and human validation. We further prune the resulting set of labelled hashtags by retaining only those that meet the following criterion.

$n_{i}>r\max_{v_{j}\in C_{m}} n_{j}$ (4)

where $n_{i}$ is the occurrences of the hashtag associated with vertex $v_{i}$, $C_{m}$ is the label of $v_{i}$ and $r=0.001$ is a threshold parameter. Finally, discovered hashtags are human validated to select hashtags having direct reference to the candidate, its party or slogans of the candidate and that express an opinion. In practice, the entire process (steps 1-5) is iterative until a stable set of hashtags is found. Table 1 shows the top-10 labelled hashtags with the highest occurrence for each category. The final network results in four different clusters corresponding to the Pro-Clinton, Anti-Clinton, Pro-Trump and Anti-Trump hashtags (Fig 1).

Table 1 Top-10 labelled hashtags with the highest occurrence for each category

| Pro-Clinton | Anti-Clinton | Pro-Trump | Anti-Trump |
| --- | --- | --- | --- |
| #imwithher | #dickileaks | #trumppence16 | #dumpthetrump |
| #clintonkaine | #hillaryforprison2016 | #trumprally | #nevertrump |
| #hillary2016 | #neverhillary | #trumpstrong | #lockhimup |
| #votehillary | #crookedhillary | #draintheswamp | #bullytrump |
| #imwithher2016 | #hillarysemails | #maga3x | #nastywomenunite |
| #hillaryforamerica | #lockherup | #maga | #racist |
| #hillaryclintonforpresident | #corrupthillary | #trump2016 | #stoptrump |
| #hillarysarmy | #clintonscandals | #votetrump | #loserdonald |
| #momsdemandhillary | #indicthillary | #makeamericagreatagain | #lyingtrump |
| #womenvote | #notwithher | #americafirst | #anybodybuttrump |


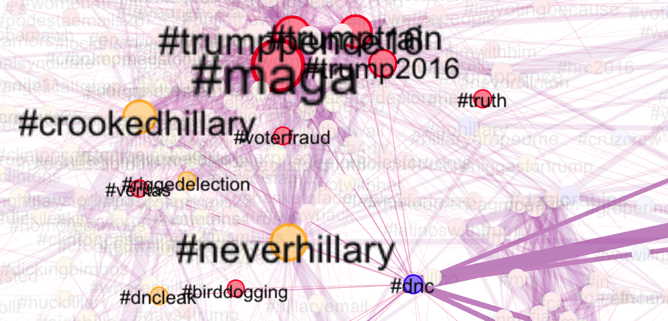

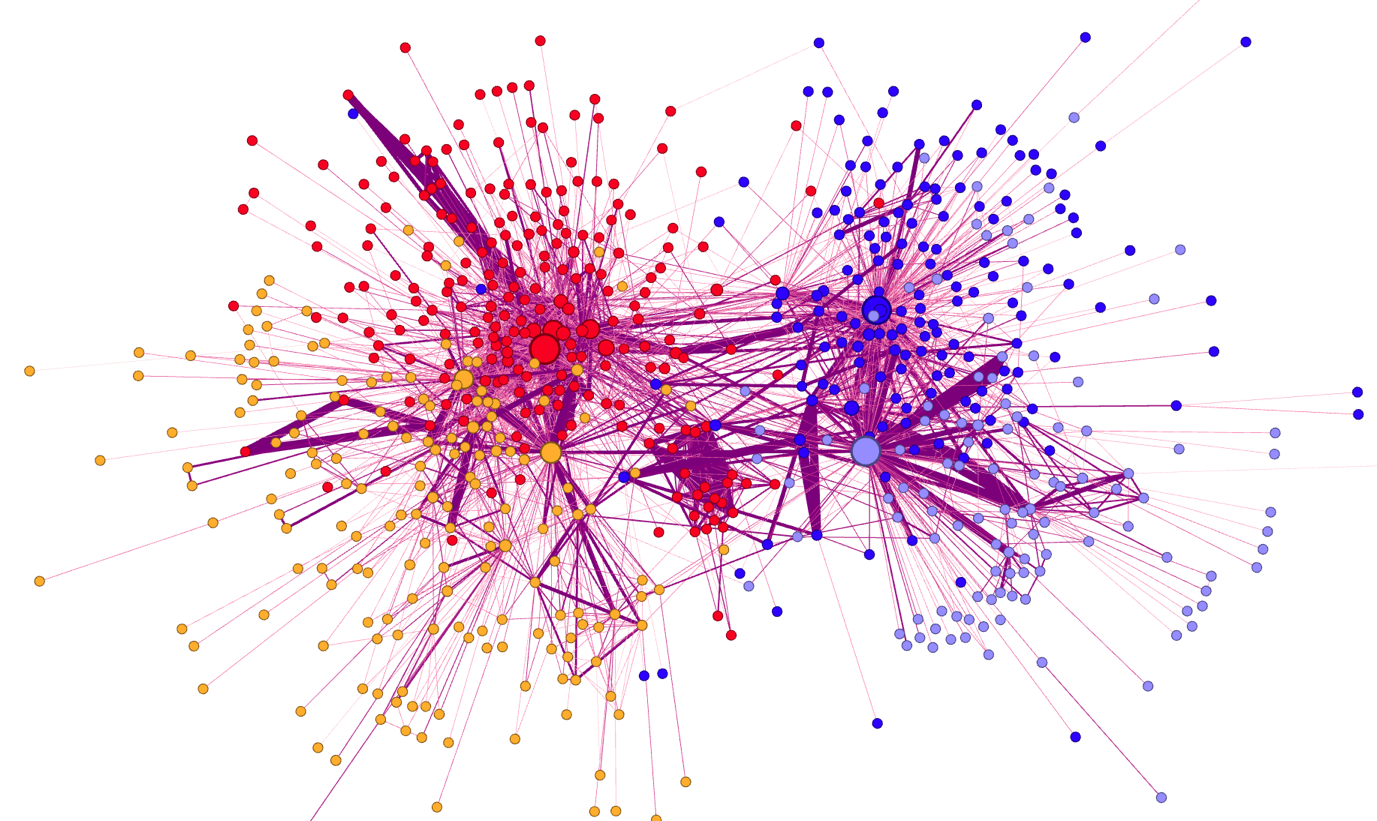


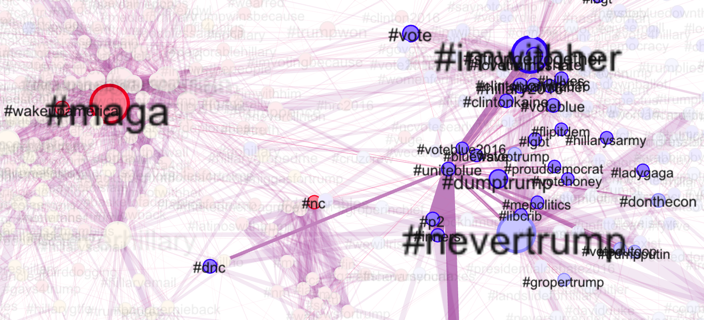


**Fig 1. Clusters of hashtags.** Red: Pro-Trump, Orange: Anti-Clinton, Dark Blue: Pro-Clinton, Light Blue: Anti-Trump.

Once a set of labelled hashtags is generated, we use them to classify tweets into opinion categories by counting number of hashtags for each label and assign the most common label to the tweet. The following 8 opinion categories are generated: Pro-Clinton, Anti-Trump, Support-Clinton (with Pro-Clinton and Anti-Trump two most common labels), Pro-Trump, Anti-Clinton, Support-Trump (with Pro-Trump and Anti-Clinton two most common labels), mixed (with one of Pro-Clinton and Anti-Trump and one of Pro-Trump and Anti-Clinton as two most common labels), and unidentified (no labelled hashtag). We use only tweets for the first 6 categories, in total 238,142, for training.

**B. Relative opinion measure at the word and user levels**

As the opinion-oriented word embedding measures relative opinion at the word level, we examine the trained embeddings for the labelled hashtags to evaluate model performance at the word level. Fig. 2 plots a two-dimensional Multi-Dimensional Scaling (MDS) representation of the hashtags in Table 1. The four seed hashtags (#maga for pro-Trump, #imwithher for pro-Hillary, #nevertrump for anti-Trump, and #neverhillary for anti-Clinton) highlighted by black circles showing a bipolarized pattern. Fig. 2 shows that the two groups, Pro-Trump/Anti-Clinton and the Pro-Clinton/Anti-Trump, of hashtags are clearly separated from one another (the dash line shows a rough dividing boundary for the two groups), while within each group the two categories of hashtags are mixed together. It indicates that our trained embeddings correctly perform with learned opinion orientations.

Furthermore, the relative opinion measure at the user level can be obtained by aggregating word embeddings first to the tweet document level then to the user level. To evaluate the performance of user-level relative opinion measure, we selected the user accounts for the two candidates, Donald Trump and Hillary Clinton, whose opinion orientations are publicly known. Fig. 3 shows a two-dimensional MDS representation of the aggregated opinion embeddings for the two candidates and the word embeddings of the four seed hashtags as references. The four seed hashtags are bipolarized between Pro-Trump/Anti-Clinton and the Pro-Clinton/Anti-Trump, as they are trained embeddings (in-sample prediction). The two candidates’ user accounts can be reasonably separated and they are toward the expected opinion orientations respectively according to their distances to the four opinion extremes identified by the seed hashtags. As the two candidates’ opinion embeddings are inferred (out-of-sample prediction), their separation is not as extreme as the bipolarization of seed hashtags. Rather, they show some similarity, because both user accounts have posted tweets that include hashtags belonging to the opposing opinion categories for the purpose of attack or criticizing.


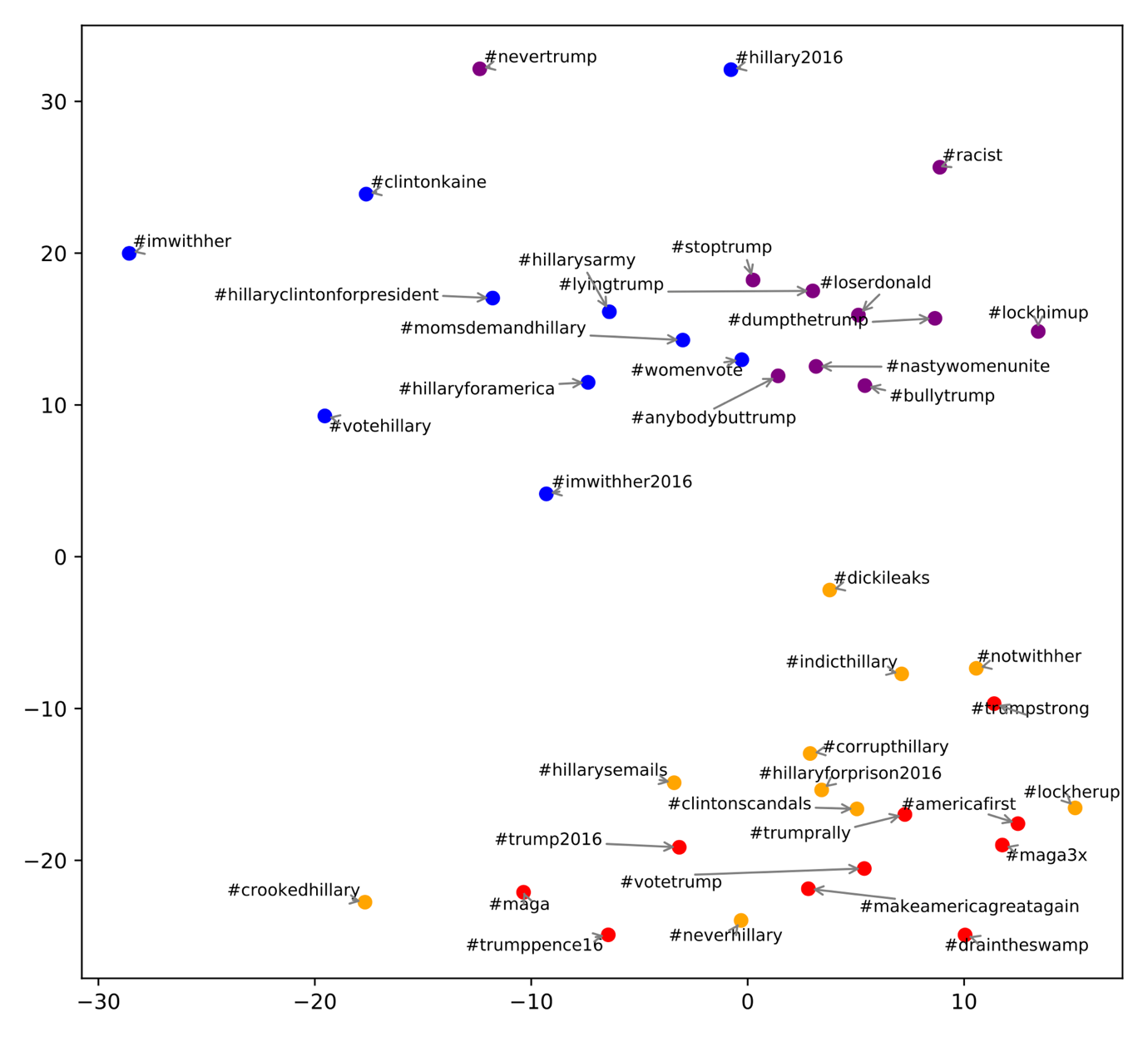


**Fig 2. Two-dimensional MDS representation of the word embeddings for the hashtags in Table 1.** Red: Pro-Trump, Orange: Anti-Clinton, Blue: Pro-Clinton, Purple: Anti-Trump.


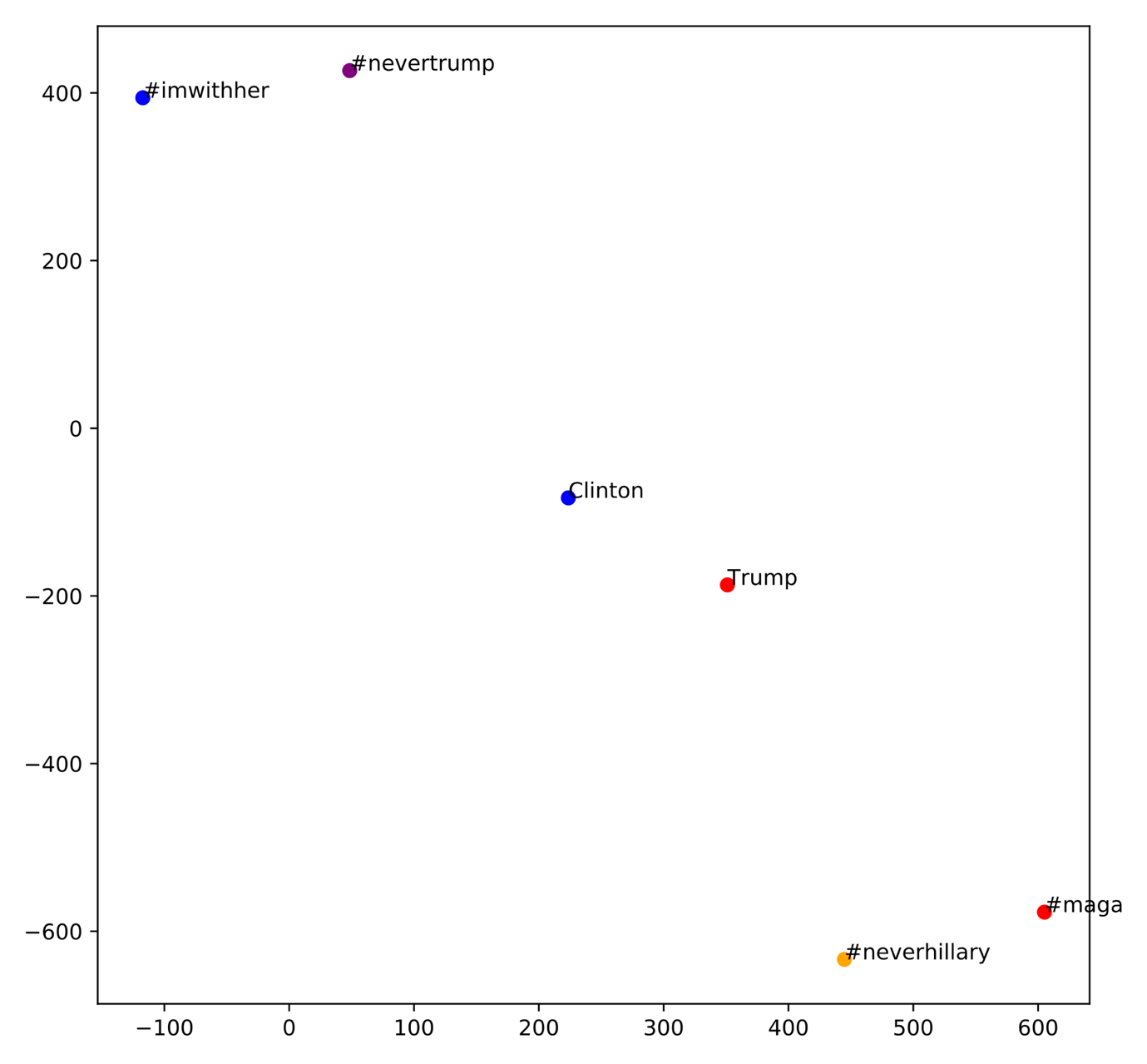
 **Fig 3. Clusters of hashtags.** Red: Pro-Trump, Orange: Anti-Clinton, Blue: Pro-Clinton, Purple: Anti-Trump.

References

1. Martinez-Romo J, Araujo L, Borge-Holthoefer J, Arenas A, Capitán JA, Cuesta JA. Disentangling categorical relationships through a graph of co-occurrences. Phys Rev E. 2011;84: 046108. doi:10.1103/PhysRevE.84.046108

2. Bovet A, Morone F, Makse HA. Validation of Twitter opinion trends with national polling aggregates: Hillary Clinton vs Donald Trump. Sci Rep. 2018;8: 8673. doi:10.1038/s41598-018-26951-y

3. Raghavan UN, Albert R, Kumara S. Near linear time algorithm to detect community structures in large-scale networks. Phys Rev E. 2007;76: 036106. doi:10.1103/PhysRevE.76.036106
